# Rationale and Design of the “DIagnostic and Prognostic Precision Algorithm for behavioral variant Frontotemporal Dementia” (DIPPA-FTD) Study: A Study Aiming to Distinguish Early Stage Sporadic FTD from Late-Onset Primary Psychiatric Disorders

**DOI:** 10.3233/JAD-230829

**Published:** 2024-01-16

**Authors:** Sterre C.M. de Boer, Lina Riedl, Chiara Fenoglio, Ishana Rue, Ramon Landin-Romero, Sophie Matis, Zac Chatterton, Daniela Galimberti, Glenda Halliday, Janine Diehl-Schmid, Olivier Piguet, Yolande A.L. Pijnenburg, Simon Ducharme

**Affiliations:** a Alzheimer Center Amsterdam, Neurology, Vrije Universiteit Amsterdam, Amsterdam UMC location VUmc, Amsterdam, The Netherlands; b Amsterdam Neuroscience, Neurodegeneration, Amsterdam, The Netherlands; c School of Psychology and Brain & Mind Centre, The University of Sydney, Sydney, Australia; d Department of Psychiatry and Psychotherapy, School of Medicine, Technical University of Munich, Munich, Germany; e Department of Biomedical, Surgical and Dental Sciences, University of Milan, Milan, Italy; f Department of Psychiatry, Douglas Mental Health University Institute, McGill University, Montreal, Canada; g Faculty of Medicine and Health, School of Health Sciences & Brain and Mind Sciences, The University of Sydney, Sydney, Australia; h Brain and Mind Centre and Faculty of Medicine and Health School of Medical Sciences, The University of Sydney, Camperdown, NSW, Australia; i University of Milan, Milan, Italy; j Fondazione Ca’ Granda, IRCCS Ospedale Maggiore Policlinico, Milan, Italy; k School of Medical Sciences & Brain and Mind Sciences, Faculty of Medicine and Health, The University of Sydney, Sydney, Australia; l kbo-Inn-Salzach-Klinikum, Clinical Center for Psychiatry, Psychotherapy, Psychosomatic Medicine, Geriatrics and Neurology, Wasserburg/Inn, Germany; m McConnell Brain Imaging Centre, Montreal Neurological Institute, McGill University, Montreal, Canada

**Keywords:** Alzheimer’s disease, diagnostics, frontotemporal dementia, neurodegeneration, prognostics, psychiatric disorders

## Abstract

**Background:**

The behavioral variant of frontotemporal dementia (bvFTD) is very heterogeneous in pathology, genetics, and disease course. Unlike Alzheimer’s disease, reliable biomarkers are lacking and sporadic bvFTD is often misdiagnosed as a primary psychiatric disorder (PPD) due to overlapping clinical features. Current efforts to characterize and improve diagnostics are centered on the minority of genetic cases.

**Objective:**

The multi-center study DIPPA-FTD aims to develop diagnostic and prognostic algorithms to help distinguish sporadic bvFTD from late-onset PPD in its earliest stages.

**Methods:**

The prospective DIPPA-FTD study recruits participants with late-life behavioral changes, suspect for bvFTD or late-onset PPD diagnosis with a negative family history for FTD and/or amyotrophic lateral sclerosis. Subjects are invited to participate after diagnostic screening at participating memory clinics or recruited by referrals from psychiatric departments. At baseline visit, participants undergo neurological and psychiatric examination, questionnaires, neuropsychological tests, and brain imaging. Blood is obtained to investigate biomarkers. Patients are informed about brain donation programs. Follow-up takes place 10-14 months after baseline visit where all examinations are repeated. Results from the DIPPA-FTD study will be integrated in a data-driven approach to develop diagnostic and prognostic models.

**Conclusions:**

DIPPA-FTD will make an important contribution to early sporadic bvFTD identification. By recruiting subjects with ambiguous or prodromal diagnoses, our research strategy will allow the characterization of early disease stages that are not covered in current sporadic FTD research. Results will hopefully increase the ability to diagnose sporadic bvFTD in the early stage and predict progression rate, which is pivotal for patient stratification and trial design.

## INTRODUCTION

The behavioral variant of frontotemporal dementia (bvFTD) is a common cause of early-onset dementia with heterogeneity in underlying pathology, genetics, and natural disease course [1–3]. Early bvFTD can manifest with neuropsychiatric symptoms such as psychosis, mood- and obsessive compulsive disorder like symptoms [4] and is therefore often mistaken for a primary psychiatric disorder (PPD) [5]. This symptomatic overlap causes substantial diagnostic delay ranging between 3 and 6 years on average [6–8]. Even once a diagnosis of bvFTD has been established, clinicians currently lack the appropriate tools to predict the rate of progression of the disease, leading to considerable uncertainty and anxiety for patients and families [9].

Recent research attention has been on genetic FTD, mostly accounted for by pathogenic mutations in the genes microtubule associated protein tau (*MAPT*), progranulin (*GRN*), and chromosome 9 open reading frame 72 (*C9orf72*) [10, 11]. However, the critical challenge is how to diagnose non-familial forms of bvFTD, also known as sporadic bvFTD [12], which accounts for 80% of all cases. Unlike with Alzheimer’s disease or genetic FTD, reliable *in vivo* biomarkers for sporadic FTD are not available, making the distinction between sporadic bvFTD and late-onset psychiatric disorders even more challenging [13]. Current clinical tools including structural/functional imaging and neuropsychology have major limitations, and emerging blood-based neurofilament light and glial fibrillary acidic protein levels hold promise but require further validation [14, 15]. There are no established fluid biomarkers for FTD yet. In fact, a certain bvFTD diagnosis of a sporadic case, classified as ‘definite bvFTD ’ as per clinical criteria [16], can only be accomplished by a brain biopsy or postmortem confirmation of FTD pathology of TDP-43, TAU or FUS [2]. To this day, it is impossible to predict the underlying FTD pathology in sporadic bvFTD cases.

Identification of the earliest stages of sporadic bvFTD is urgently needed. These early sporadic cases would be the ideal target for disease modifying clinical trials. With the development of disease-modifying therapeutics, it is becoming increasingly important to be able to target patients in the earliest stage of bvFTD. However, given the nature of initial symptoms that overlap with PPD, clinicians cannot reliably identify an equivalent to the mild cognitive impairment in subjects who do not carry a pathogenic mutation. This seriously limits research in this field. In addition, an ante-mortem prediction of FTD pathology is required, in order to plan precision medicine trials according to the mode of action of new compounds.

As a response to these major problems, the multi-center study called “DIagnostic and Prognostic Precision Algorithm for behavioral variant Frontotemporal Dementia” (DIPPA-FTD) was established. The DIPPA-FTD study has combined cohorts from several countries into a comprehensive retrospective discovery cohort focusing on sporadic cases and PPD, in addition to a large number of pathologically confirmed bvFTD and PPD cases. The retrospective study has identified several promising clinical and biological markers to distinguish bvFTD and PPD. In the DIPPA-FTD prospective study, clinically less well-defined bvFTD and PPD cases are being deeply phenotyped and studied to validate these promising markers. The inclusion of subjects with uncertain/ambiguous diagnoses that might turn out to be primary psychiatry or bvFTD is an innovative strategy that allows the investigation of the earliest stages of sporadic bvFTD. We will use statistical modelling to create the best diagnostic and prognostic model for sporadic bvFTD and PPD. As a sub-goal, we aim to determine underlying FTD pathology within the sporadic bvFTD group, to enable eventual patient stratification. The DIPPA-FTD study will contain the first international initiative on sporadic bvFTD and late-onset psychiatric disorders. In this paper, we will explain the design of the prospective DIPPA-FTD study.

## METHODS

### Study structure

DIPPA-FTD is a multi-center prospective cohort study based at the Alzheimer center Amsterdam, Brain and Mind Centre of the University of Sydney, the Douglas Mental Health University Institute (McGill University), Fondazione Ca’ Granda, IRCCS Ospedale Maggiore Policlinico, University of Milan, and the Technical University of Munich. The study recruits participants with late-life behavioral changes, suspect for bvFTD or late-onset PPD diagnosis with a negative family history for FTD and/or amyotrophic lateral sclerosis or with documented negative genetic testing. DIPPA-FTD study participants all undergo a baseline assessment, including a neuropsychological test battery, patient and caregiver questionnaires, blood draw and a research MRI scan of the brain. This assessment is repeated after one year at a follow-up visit. The diagnostic and prognostic value of the assessment and each test is compared to the gold standard, which is the longitudinal diagnosis from expert clinicians.

### Ethics statement

The study has been approved by the local ethical committee from each participating site.

### Study participants

#### In- and exclusion criteria

The DIPPA-FTD study includes subjects with late-life behavioral changes after the age of 45 years. Subjects meeting the Rascovsky criteria for possible or probable bvFTD, and subjects meeting one of the DSM-V classifications major depressive disorder, manic episode, bipolar disorder, schizophrenia, schizotypal disorder, delusional disorder, or obsessive-compulsive disorder (OCD) are included. Subjects not completely fulfilling Rascovsky criteria or the above-mentioned DSM-V diagnosis at baseline will remain in the study as ‘ambiguous cases’; this is to prevent an exclusion of a possibly early sporadic bvFTD case. The study aims to include *n* = 100 subjects with sporadic bvFTD cases and *n* = 100 subjects with a primary psychiatric disorder. Ambiguous subjects are distributed based on the most likely etiology (psychiatric or FTD based on a five-point Likert scale). Subjects with a positive psychiatric history before the age of 45 can still be enrolled for the study on the condition that the early-life psychiatric history is unrelated to the current late-onset behavioral change and is relatively mild (e.g., a patient with late-onset psychosis reporting a mild depressive episode in a situational context in early adulthood).

Subjects are recruited at participating memory clinics or via referrals from the psychiatry department. All subjects undergo a comprehensive diagnostic screening, following local guidelines of the participating memory clinics, to rule out the existence of an underlying Alzheimer’s disease or other neurological disorders. For each subject, the family history is reviewed before inclusion. Subjects with a Goldman score of≥3 [17] or a Wood score of ‘Low’ or ‘Apparent sporadic’ [18] are included if *C9orf72* repeat expansion screening is negative. Subjects with a Goldman score of 2 or a Wood score of ‘Medium’ are included if genetic testing of *C9orf72, GRN*, and *MAPT*, the three most prevalent pathogenic mutation in FTD, are all negative. Subjects with a Goldman score 1 and Wood score ‘High’ are excluded. Other exclusion criteria are a Mini-Mental State Examination score below 18 points, Clinical Dementia Rating (CDR) scale of≥2, positive Alzheimer’s disease biomarkers, insufficient comprehension of the assessment language, the absence of a reliable informant or the presence of a causal pathogenic FTD mutation.

### Study procedures

**Fig. 1:**
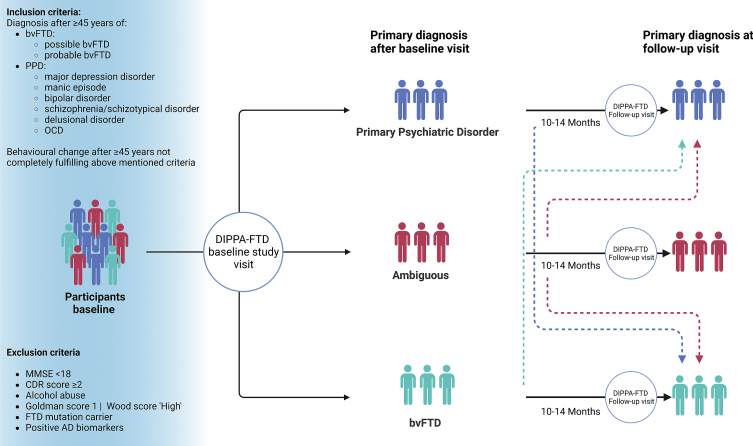
Visual representation of the study visits of the DIPPA-FTD study (created with BioRender.com). bvFTD, behavioral variant of frontotemporal dementia; PPD, primary psychiatric disorder; OCD, obsessive compulsive disorder; MMSE, Mini-Mental State Examination; CDR, Clinical Dementia Rating; AD, Alzheimer’s disease.

**Fig. 2:**
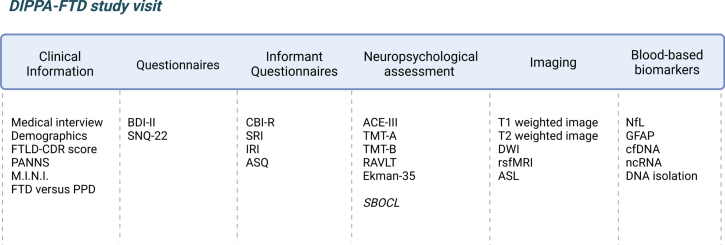
Overview study protocol of prospective DIPPA-FTD study (created with BioRender.com). FTLD-CDR score, frontotemporal Lobar degeneration Clinical Dementia Rating scale; PANNS, Positive And Negative Syndrome Scale; M.I.N.I., Mini-International Neuropsychiatric Interview; BDI-II, Beck Depression Inventory®-II; SNQ-22, Social Norm Questionnaire-22; CBI-R, Cambridge Behavioral Inventory Revised; SRI, Stereotypy Rating Inventory; IRI, Interpersonal Reactivity Index; ASQ, Autonomic Symptoms Questionnaire; ACE-III, Addenbrooke’s Cognitive Examination III; TMT-A, Trail Making Test part A; TMT-B, Trail Making Test part B; RAVLT, Rey Auditory Verbal Learning Test; SBOCL, Social Behavior Observer Checklist; DWI, Diffusion Weighted Imaging; rsfMRI, resting state functional MRI; ASL, arterial spin labelling; NfL, neurofilament light; GFAP, glial fibrillary acidic protein; cfDNA, cell free DNA; ncRNA, non-coding RNA.

An overview of the in- and exclusion criteria and the study visit structure is shown in Fig. 1. All tests, questionnaires, and neuropsychological test battery used in the DIPPA-FTD study were available and validated in Dutch, English, French, German, and Italian unless other indicated. All information and data obtained in the DIPPA-FTD study is stored in a secured Castor EDC database. An overview of the study protocol is shown in Fig. 2.

### Clinical information

At each study visit, the participants undergo a structured medical interview by a trained physician to obtain data on demographics, neurological and psychiatric symptoms, family history information, and medication use. Neurological examination is performed. If the participants give consent, their informant is also interviewed. In addition, several bedside tools and questionnaires are measured by the physician:

– The *FTLD-CDR score* is rated after the structured medical interview by the physician to register disease severity.
– The *Positive And Negative Syndrome Scale (PANNS)* is a commonly used scale that quantify psychotic symptoms [19]. On item level, the PANNS have shown to differ between PPD and bvFTD patients [20]. In this study, the PANSS is used to systematically assess the presence of psychotic–like symptoms.
– The *Mini-International Neuropsychiatric Interview (M.I.N.I.)* [21] was developed to provide a structured psychiatric interview. Here, the physician conducts their own psychiatric assessment and uses the first page of the M.I.N.I to improve consistency of the psychiatric diagnostic reporting between centers.
– The ‘*FTD versus PPD*’ bedside tool is a clinical characteristics checklist of 17 items that can help the clinician to distinguish FTD from PPD [22]. Each question is answered by yes or no and corresponds to an indication of bvFTD or PPD with a score of≥11 being indicative of bvFTD. The tool is filled out by the physician after the clinical evaluation of the DIPPA-FTD study.

### Subjective questionnaires

Two questionnaires are either self-administered or verbally administered by a trained researcher or neuropsychologist. The *Beck Depression Inventory*®*-II* (BDI®-II) is used to assess the presence and severity of depression in the DIPPA-FTD participant. The BDI-II contains 21 mood-related statements, the participant is asked to pick the statement that describes his/her mood of the last 2 weeks, including the day of administration [23, 24]. The *Social Norm Questionnaire-22 (SNQ-22*) is administered to measure comprehension of widely accepted social norms [25]. The participant is presented 22 social situations and asked whether or not it is socially acceptable and appropriate to do these things in his/her culture and answers yes or no to each one. A total score and two error scores are derived, giving an estimation of comprehension of social norms as well as a tendency to break or to be overly adherent.

### Informant questionnaires

The informant (e.g., partner, sibling, primary caregiver) of the participant are requested, with consent of the participant, to complete questionnaires related to the behavior of the participant. The questionnaire is administered by a trained reseacher via an interview. The following questionnaires are administered:

– The *Cambridge Behavioral Inventory Revised (CBI-R)* is a 45-item questionnaire to determine the frequency and severity of the neuropsychiatric and psychiatric symptoms in neurodegenerative disorders [26]. Throughout 10 different domains with several sub-items, the informant is asked if he/she noticed any difference in memory & orientation, everyday skills, self-care, abnormal behavior, mood, beliefs, eating habits, sleep, stereotypic motor & behaviors and motivation. The researcher tests whether the informant noticed any difference and if so, the frequency and severity of each sub-item is rated.
– The *Stereotypy Rating Inventory (SRI)* measures 5 distinctive stereotypical behavioral disturbances that are often present in FTD cases: A) Eating and cooking behaviors, B) Roaming, C) Speaking, D) Movements, and E) Daily rhythm [27]. The researcher determines the frequency and severity of each behavior, similar to the CBI-R.
– The *Interpersonal Reactivity Index (IRI)* is a measure of the cognitive and emotional components of empathy [28]. For this study, the adjusted “co-participant” questionnaire is used. This version, provided by the National Alzheimer’s Coordinating Center, has 14 statements, on ‘perspective takings’ and ‘empathic concern’, answered on a 5-point Likert scale from ‘Does not describe well (1)’ to ‘Describes very well (5)’. The informant is asked how well each statement describes the participants current behavior.
– The *Autonomic symptoms questionnaire (ASQ)* monitors the presence and frequency of autonomic symptoms, often reported in bvFTD [29, 30]. The questionnaire is divided in 5 sections: 1) blood pressure, 2) gastrointestinal symptoms, 3) temperature regulation and sweating, 4) urinary symptoms, and 5) sleeping symptoms [30]. For the DIPPA-FTD study, a sixth section was added, and translated, on pain perception (Supplementary Material S1). Carers are asked to rate the frequency of autonomic symptoms on a 5-point Likert scale, ranging from 0 (never) to 5 (daily) and the severity on a 3-point scale, ranging from 0 (not applicable) to 3 (severe) for each symptom over the past 6 months. After which a composite score of frequency x severity is calculated.

### Neuropsychological assessment

In the DIPPA-FTD study, the *Addenbrooke’s Cognitive Examination III (*ACE-III) is administered to screen global cognitive functioning. The ACE-III is a commonly used cognitive screening tool and tests six separate domains with a maximum score of 100: orientation (10), attention (8), memory (35), verbal fluency (14), language (28), and visuospatial ability (5) [31, 32]. The ACE-III was already available in each primary DIPPA-FTD site’s language with the exception of Dutch, for which the Flemish ACE-III was used for translation. In addition to the ACE-III, all participants undergo a short neuropsychological test battery that includes:

– The *Trail Making Test part A and part B* (TMT-A and TMT-B respectively) to test processing speed, sequencing, mental flexibility and visual-motor skills.
– The *Rey Auditory Verbal Learning Test* (RAVLT) to test verbal memory. Participants are given a list of 15 unrelated words repeated over five different trials and are asked to repeat. Next, another list of 15 unrelated words are given and the participant must repeat the original list of 15 words. The latter is repeated after 30 minutes. The Dutch RAVLT was adjusted to bring the test into line with each site (Supplementary Material S2).
– The *Ekman-35 test* to assess face emotion recognition as measurement of social cognition. The Ekman-35 test is derived from the Face Emotion Recognition task in the GENFI protocol [33] and is a shortened version of the standard Ekman Face test [34]. The participant is presented 35 faces, 5 faces per emotion: 1) happiness, 2) surprise, 3) anger, 4) fear, 5) disgust, and 6) sadness, and is asked to label the emotion of the face presented.

After the neuropsychological assessment, the examiner completes the *Social Behavior Observer Checklist (SBOCL)* in which the behavior of the participant during the neuropsychological testing is evaluated [35]. The checklist is reproduced and translated with permission of the author, Katherine Rankin.

### Imaging

All participants will undergo an structural MRI with structural 3D T1 (1.1 mm isometric), T2 (1.1 mm isometric), diffusion weighted MRI (DWI –2.5 mm isometric, TR 7300 ms, TE 90.0 ms) and resting state functional MRI (rsfMRI - Voxel size: 3.0×3.0×3.5 mm, 8 : 29 minute acquisition) at baseline and at follow-up. Acquisition time is around 35 minutes, depending on the scanner.

### Blood-based biomarkers

At each site, venous blood is drawn at baseline and at follow-up. Within 2 hours after collection, plasma and serum is derived by centrifugation at 1800-1900 g, for 10-15 minutes at room temperature. PAXgene Blood ccfDNA tubes are centrifuged at room temperature (15-25°C) for 15 minutes at 1900 g to isolate blood plasma and buffy coat. Samples are stored at -80°C at each site till further analysis. Serum samples will be used to determine neurofilament light and glial fibrillary acidic protein levels. Plasma will be used to determine brain-derived cfDNA levels, as described in Chatterton et al., 2021 [36], and also ncRNA levels in Neural Derived Exosomes as described in Serpente et al., 2020 [37] and Visconte et al., 2023 [38]. DNA will be isolated and will be used to identify genetic risk factors.

### National brain bank programs

Australian and Dutch participants are made aware of the possibility to register for local brain donation programs; the Sydney Brain Bank (https://www.neura.edu.au/scientific-facility/sydneybrainbank/) and the Netherlands Brain Bank (https://www.brainbank.nl) respectively during the study visits. Once enough pathological data becomes available of DIPPA-FTD participants, the collected data of the prospective study can be used in a retrospective manner to run logistic regression models to predict underlying FTD pathology, which will enable patient stratification in the future.

### Diagnostic procedure

The DIPPA-FTD participant will be given a primary diagnosis (bvFTD, ambiguous, or PPD) and its diagnostic subtype after clinical evaluation is completed. The physician is asked to rate the certainty of the given primary diagnosis ranging from: confident bvFTD, probably bvFTD, unsure if bvFTD or PPD, probably PPD, confident PPD. In addition to the primary diagnosis, a differential diagnosis is made. This diagnostic procedure is repeated at the follow-up visit. The expert diagnosis given at the follow-up visit is acknowledged as the ‘gold standard’.

### Statistics

While individual markers (clinical information, neuroimaging, blood-based biomarkers) have potential diagnostic and prognostic value, data-driven integration of all relevant variables into a united model will truly enable personalized medicine. The objective is to find the simplest algorithm of testing that will optimize diagnostic accuracy. If accuracy over 95% cannot be obtained with a combination of 2-3 tests, we will explore if integrating a larger number of features could lead to improved accuracy. This would include integrating all biological, imaging, blood, and clinical variables into a machine learning (e.g. random forest) classifier to identify, in a data-driven way, subjects with PPD versus bvFTD. This would be performed with 90% of the cohort, leaving 10% of subjects as an independent test set. Once sufficient pathology is obtained, a similar process will be performed to predict subtypes of FTLD pathology.

This data will be used to create diagnostic algorithms to accurately differentiate between sporadic bvFTD and PPD in a cohort with individuals presenting with late-onset behavioral change. Within the sporadic bvFTD group, pathological data, once available, will be used to predict underlying pathology.

## DISCUSSION

This design paper provides detailed information about the aim, rationale, and structure of the DIPPA-FTD study. This prospective cohort study has the aim to develop diagnostic and prognostic algorithms to distinguish sporadic bvFTD from late-onset PPD and to provide a timely diagnosis and clinical care for patients with adult-onset behavioral changes.

The difficulty of distinguishing sporadic bvFTD from late-onset PPD endures with the lack of reliable (non-genetic) FTD biomarkers. Even though sporadic FTD comprises the majority of all FTD cases, it has been understudied in comparison to genetic FTD. In particular, there has been major interest to clarify the prodromal biological changes leading to the onset of FTD using genetic mutation carriers as a model. Proposed definitions of mild cognitive behavioral impairment in FTD are predominantly based on genetic samples, given the small number of patients that have autopsy confirmation and early-stage characterization [39]. This is in part because current cohort studies of sporadic FTD enroll patients once a diagnosis is present, thereby excluding patients with ambiguous early stage of the disease when subtle psychiatric features predominate.

In response, studies focusing on late-onset behavioral disorders have gotten increasing attention, such as the Late-onset Frontal lobe syndrome study [40–42] and the Neuropsychiatric International Consortium for FTD (NIC-FTD) [13]. However, the DIPPA-FTD study is the first international consortium prospectively studying sporadic FTD and its differentiation from late-onset PPD, therefore considered one of its biggest strengths. The choice of clinical, neuropsychological, and biomarker assessment has been mostly inspired by NIC-FTD’s clinical recommendations to distinguish frontotemporal dementia from psychiatric disorders [13] and the retrospective DIPPA-FTD study (de Boer et al, in preparation). In order to maintain and improve translational feasibility and efficiency, we decided not to implement all the recommended tests by NIC-FTD into our protocol. With that said, we have purposely chosen to pursue several clinical variables, neuropsychological data, blood-based markers and imaging variables for investigation that can also be compared to data from genetic FTD cohorts such as the Genetic Frontotemporal dementia Initiative (GENFI) study [43], ALLFTD [44], and other dementia cohorts.

This study has several challenges. Even though this is a prospective study with a uniform protocol across each site, the different health care systems and its referral procedures cannot be completely harmonized which might lead to inclusion bias. Likewise, sociocultural factors can differ between sites and are known to affect performance on social cognitive and behavioral tests [45] which should be taken into account when interpreting results. On the other hand, the ethnic background of the populations from Australia, Canada, and Western Europe are considered quite similar due to the history of human migration. This raises the question about reproducibility in populations from different ethnic and cultural backgrounds. Therefore, within the DIPPA-FTD study, we will document each participant’s ethnic background to reflect on our representability of local populations. This also gives the unique opportunity to study potential transcultural variability in our results. Future directions include the participation of sites from different ethnic, economic, and cultural backgrounds to increase diversity and reproducibility. Second, only the Australian and Dutch site have a national brain donation program, thus a pathological verification in the future will only be available for a small proportion of the samples, unless further research funding is obtained to perform research autopsies. Lastly, over a one-year follow-up, some patients might remain with an ambiguous diagnosis; however, the consortium intends to follow diagnostic evolution over the long-term, mimicking the approach in long-term genetic cohorts. In addition, modelling disease progression of these uncertain cases can still be of value and provide a worthy reflection of a neuropsychiatric cohort.

### Conclusion

The results of the DIPPA-FTD study will increase the ability to reliably identify sporadic bvFTD cases in their early prodromal stages and aims to predict rate of progression in sporadic bvFTD and other patients presenting with behavioral changes. The consortium innovates by integrating patients with bvFTD, ambiguous diagnoses and late-onset psychiatric disorders, which are often the main differential diagnosis, into a single prospective cohort. This research aims to complement current efforts in genetic FTD by targeting sporadic cases for patient stratification, trial design and personalized treatments.

## Supplementary Material

Supplementary Material

## Data Availability

The data resulting from this study will be available upon reasonable request from the corresponding author. The study protocol is available upon request.
