# Supplementary material for: Rationale and Design of the “DIagnostic and Prognostic Precision Algorithm for behavioral variant Frontotemporal Dementia” (DIPPA-FTD) Study: A Study Aiming to Distinguish Early Stage Sporadic FTD from Late-Onset Primary Psychiatric Disorders
Source: J Alzheimers Dis. 2024 Jan 16;97(2):963–73. doi: 10.3233/JAD-230829 (PMC10836537; doi:10.3233/JAD-230829)
Supplement: Supplementary Material [file jad-97-jad230829-s001.pdf]

Supplementary Material

Rationale and Design of the "DIagnostic and Prognostic Precision Algorithm for behavioral variant Frontotemporal Dementia" (DIPPA-FTD) Study: A Study Aiming to Distinguish Early Stage Sporadic FTD from Late-Onset Primary Psychiatric Disorders

S1. Section 6 on pain perception in the Autonomic Symptoms Questionnaire for the DIPPA-FTD study

Section 6: pain perception.

**45. Does he/she reported strange unexplained physical complaints?**						
**A.**	**Frequency**	Never	less than 1/ month	1-4 times/ month	more than 1/ month-	Daily
**B.**	**Severity**	--- N/A---	---Moderate to severe----	----Severe----		

**46. Is her or his perception of pain altered in comparison before the disease?**						
**C.**	**Frequency**	Never	less than 1/ month	1-4 times/ month	more than 1/ month-	Daily
**D.**	**Severity**	--- N/A---	---Moderate to severe----	----Severe----		

If yes: please describe the complaints and symptoms.

S2. Dutch version of the RAVLT for the DIPPA-FTD study

Lijst A: 15-woordentest, vorm A.
Lijst B: 15-woordentest, vorm K.

Trial A1: ik ga een lijst met woorden voorlezen. Luister goed. Als ik klaar ben met de woorden voorlezen, mag u zo veel mogelijk woorden opnoemen die u heeft onthouden. Het maakt niet uit in welke volgorde u deze woorden opnoemt.

Trial 2-5: ik ga nu dezelfde woorden nogmaals voorlezen. Als ik klaar ben met de woorden voorlezen, mag u weer zo veel mogelijk woorden opnoemen die u heeft onthouden. Het maakt niet uit als u de woorden al eerder heeft genoemd. De volgorde maakt wederom niet uit.

Trial B1 (lijst B): ik ga nu een nieuwe lijst met woorden voorlezen.

Lijst A										
	**A1**	**A2**	**A3**	**A4**	**A5**	*Lijst B*	**B1**	**A6**	**A7** (20-30 min later)	**Recall Lijst A**
BLOEM						MES				BLOEM
SNOR						BANK				SNOR
KROON						WOLF				KROON
BEER						HAARD				BEER
LAP						STOEP				LAP
KLOK						KAT				KLOK
ZWAAN						BRIEF				ZWAAN
REK						RING				REK
TAS						KRANT				TAS
BERG						PAAL				BERG
KRUIK						NEK				KRUIK
HOEN						SPEER				HOEN
GOOT						BOOT				GOOT
MAAG						TUIN				MAAG
KAR						PIJL				KAR
Intrusies										
**Correct**										

Herkenning (20-30 minuten na Lijst A en B)
(omcirkel het woord als de participant JA zegt)

"Ik ga een lijst met woorden opnoemen, een voor een. Als het woord op de eerste lijst (de lijst die ik meermaals aan u heb voorgelezen) voorkwam, dan zegt u JA. Als het woord niet op de eerste lijst voorkwam, zegt u NEE"

BROOD	NET	NAR	**BEER**	**LAP**	HAAN
**KAR**	**KLOK**	VOS	**GOOT**	**KROON**	**REK**
BEUK	**MAAG**	KEEL	**TAS**	FILM	KOE
PAN	HAK	**SNOR**	DUIM	**HOEN**	ROK
**ZWAAN**	**BLOEM**	POMP	BOEF	**BERG**	**KRUIK**

Correcte herkenning: _______

Vals positief: ______
